# Mob/*oriT*, a mobilizable site-specific recombination system for unmarked genetic manipulation in *Bacillus thuringiensis* and *Bacillus cereus*

**DOI:** 10.1186/s12934-016-0492-9

**Published:** 2016-06-10

**Authors:** Pengxia Wang, Yiguang Zhu, Yuyang Zhang, Chunyi Zhang, Jianyi Xu, Yun Deng, Donghai Peng, Lifang Ruan, Ming Sun

**Affiliations:** State Key Laboratory of Agricultural Microbiology, College of Life Science and Technology, Huazhong Agricultural University, Wuhan, People’s Republic of China

**Keywords:** *Bacillus thuringiensis*, *Bacillus cereus*, Relaxase, Mob protein, Site-specific recombination, Conjugation

## Abstract

**Background:**

*Bacillus thuringiensis* and *Bacillus cereus* are two important species in *B. cereus* group. The intensive study of these strains at the molecular level and construction of genetically modified bacteria requires the development of efficient genetic tools. To insert genes into or delete genes from bacterial chromosomes, marker-less manipulation methods were employed.

**Results:**

We present a novel genetic manipulation method for *B. thuringiensis* and *B. cereus* strains that does not leave selection markers. Our approach takes advantage of the relaxase Mob02281 encoded by plasmid pBMB0228 from *Bacillus thuringiensis*. In addition to its mobilization function, this Mob protein can mediate recombination between *oriT* sites. The Mob02281 mobilization module was associated with a spectinomycin-resistance gene to form a Mob-Spc cassette, which was flanked by the core 24-bp *oriT* sequences from pBMB0228. A strain in which the wild-type chromosome was replaced with the modified copy containing the Mob-Spc cassette at the target locus was obtained via homologous recombination. Thus, the spectinomycin-resistance gene can be used to screen for Mob-Spc cassette integration mutants. Recombination between the two *oriT* sequences mediated by Mob02281, encoded by the Mob-Spc cassette, resulted in the excision of the Mob-Spc cassette, producing the desired chromosomal alteration without introducing unwanted selection markers. We used this system to generate an in-frame deletion of a target gene in *B. thuringiensis* as well as a gene located in an operon of *B. cereus*. Moreover, we demonstrated that this system can be used to introduce a single gene or an expression cassette of interest in *B. thuringiensis*.

**Conclusion:**

The Mob/*oriT* recombination system provides an efficient method for unmarked genetic manipulation and for constructing genetically modified bacteria of *B. thuringiensis* and *B. cereus*. Our method extends the available genetic tools for *B. thuringiensis* and *B. cereus* strains.

**Electronic supplementary material:**

The online version of this article (doi:10.1186/s12934-016-0492-9) contains supplementary material, which is available to authorized users.

## Background

The *Bacillus cereus* group currently includes eight closely related species: *B. anthracis*, *B. cereus*, *B. thuringiensis*, *B. cytotoxicus*, *B. mycoides*, *B. pseudomycoides*, *B. weihenstephanensis* and *B. toyenensis* [1–3]. These microbial organisms are widespread in natural environments and have high economic, medical, and biodefense importance. *B. cereus* is a well-known food poisoning bacterium; *B. thuringiensis*, which produces insecticidal crystal proteins and is widely used to control agricultural pests [1]. The development of global systems biology tools, such as genome sequencing technologies and DNA microarrays, has stimulated the study of functional genes, biosynthetic gene clusters, and other aspects of cell biology [4, 5]. The intensive study of *B. thuringiensis and B. cereus* strains at the molecular level and construction of genetically modified bacteria requires the development of efficient genetic tools, especially vectors for targeted gene deletions and insertions.

Gene inactivation via the insertion of an antibiotic-resistance marker into a target gene is frequently used for functional analyses. However, when a single strain is manipulated repeatedly, the small number of convenient selectable markers limits the application of this approach. Moreover, since operons are common in bacterial genomes, the insertion of a marker gene can alter the expression of adjacent genes. Third, genetically modified bacteria containing antibiotic-resistance genes are not desirable in production systems, because the antibiotic resistance represents a contaminant of the final product and carries the risk of horizontal gene transfer of antibiotic-resistance genes to the environment. Therefore, a method of inserting genes into or deleting genes from bacterial chromosomes without leaving selection markers is a significant advantage. The approaches have been established in some microorganisms by the utilization of recombinase-mediated recombination systems. Various site-specific recombination systems are used, e.g., the FLP/*FRT* recombination system of *Saccharomyces cerevisiae* [6–8], the Cre/*loxP* recombination system from *Escherichia coli* bacteriophage P1 [9, 10], and the Xer/*dif* recombination system based on endogenous Xer recombinases [11, 12]. In all of these systems, two different or identical recognition sites act as the DNA substrate for the recombinase, and a single recombination site remains within the target locus. The FLP/*FRT* and Cre/*loxP* systems require many time-consuming steps, including the construction of an exogenous recombinase FLP or Cre in a replicative plasmid, the introduction of the plasmid, recombinase expression in the transformant, and elimination of the replicative plasmid [7, 10]. Moreover, the FLP/*FRT* system is not efficient in some slow-growing bacteria [8]. The Xer/*dif* system utilizes the endogenous recombinase Xer to recognize and resolve *dif* sites to excise antibiotic resistance genes. However, Xer recombinases and the relevant *dif* sites differ among bacteria, and some Xer systems include two recombinases [13], while others include one recombinase [12, 14].

Relaxase and *oriT* are critical for the transfer of both conjugative plasmids and mobilizable plasmids [15]. The origin of transfer, or *oriT* sequence, is the only component that should be provided by the mobilizable plasmid in *cis*. The relaxase, which is also called a Mob protein in a mobilizable plasmid, catalyzes the initial and final stages of conjugation. In addition, to their nicking activity necessary for mobilizing plasmids, certain relaxases can catalyze recombination between two *oriT* sequences, including NikB (R64), TrwC (R388), MobA (R1162), Mob02281 (pBMB0228), and Mob02282 (pBMB0228) [16–19]. This recombination event can mediate the deletion of a target DNA region between two *oriT* sequences. Accordingly, owing to their functions in conjugation and recombination, relaxases have been used for various purposes, e.g., to construct transfer vectors [20, 21] and for *oriT*-directed cloning of large bacterial genomic regions [22]. However, relaxase-mediated mechanisms have not been used for unmarked genetic manipulation.

In a previous study, we confirmed that Mob02281 and Mob02282 not only have mobilization ability, but also mediate the resolution and formation of plasmid pBMB0228 from *B. thuringiensis* strain YBT-1518, the mechanism is *oriT* site-specific recombination [19]. Mob02281 has a higher mobilization efficiency and recombination ability than Mob02282 [19]; accordingly, we used it to develop novel molecular tools, and demonstrated the potential application of this method to other bacterial taxa.

## Results

### Relaxase Mob02281 can transfer a target gene into *B. thuringiensis* and *B. cereus* strains

**Fig. 1:**
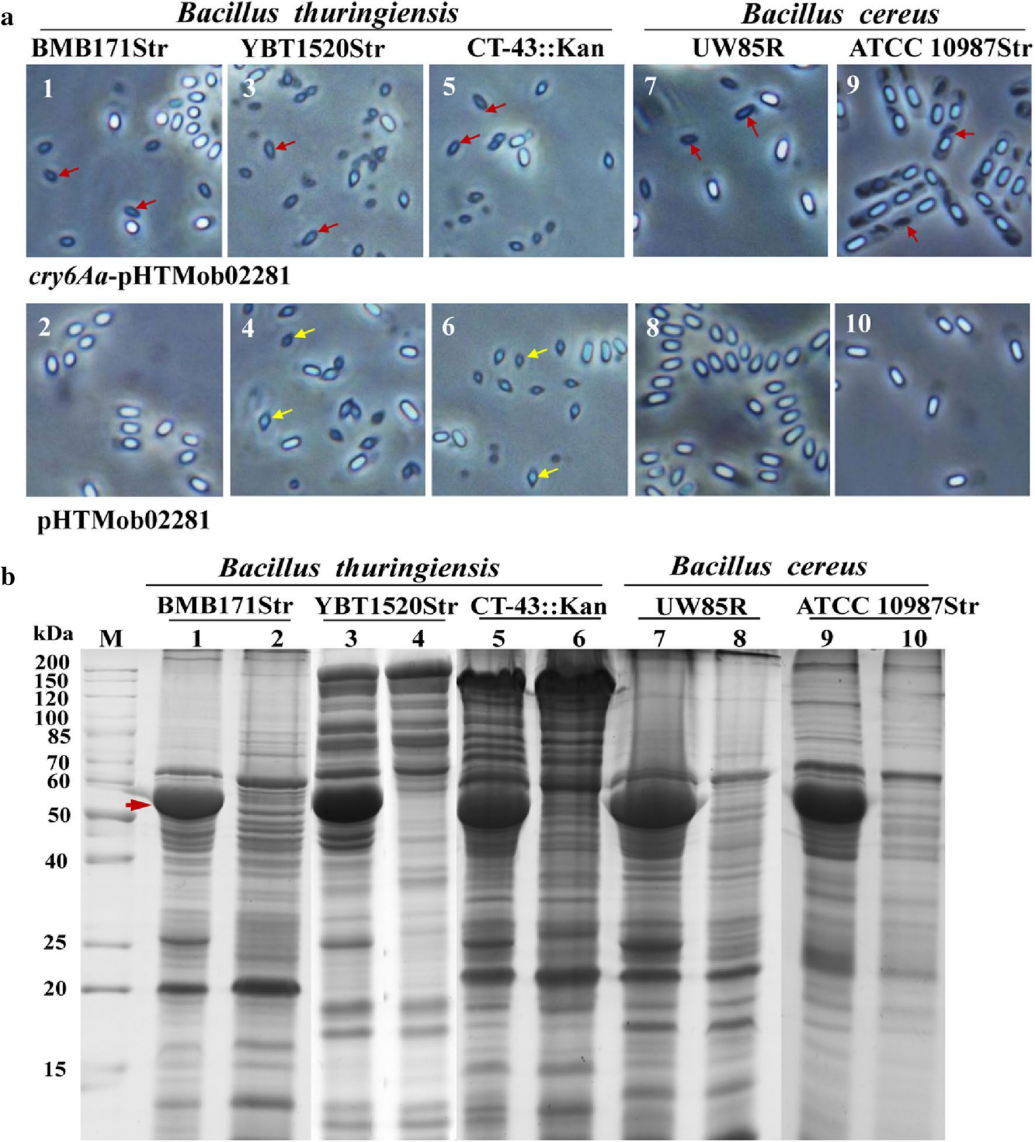
Parasporal crystals and protein expression of *cry6Aa* in transconjugants. **a** Phase-contrast microscopy image of parasporal crystals and spores produced in transconjugants. The *red arrow* indicates the *rice*-*shaped* crystals encoded by *cry6Aa*. The *yellow arrow* indicates the *diamond*-*shaped* crystals encoded by the endogenous crystal genes for YBT1520Str [5] and CT43::Kan [40]. **b** Protein analysis of the transconjugants by SDS-PAGE. *The red arrow* indicates the 54-kDa protein produced by *cry6Aa*. *1* BMB171Str/*cry6Aa*-pHTMob02281. *2* BMB171Str/pHTMob02281. *3* YBT1520Str/*cry6Aa*-pHTMob02281. *4* YBT-1520Str/pHTMob02281. *5* CT-43::Kan/*cry6Aa*-pHTMob02281. *6* CT-43::Kan/pHTMob02281. *7* UW85R/*cry6Aa*-pHTMob02281. *8* UW85R/pHTMob02281. *9* ATCC 10987Str/*cry6Aa*-pHTMob02281. *10* ATCC 10987Str/pHTMob02281. *M* Protein molecular weight marker

To test whether Mob02281 can transfer a target gene into *B. thuringiensis* and *B. cereus* strains, the plasmid *cry6Aa*-pHTMob02281, which contains *cry6Aa* as a reporter, was constructed. The *cry6Aa* gene can produce rice-shaped crystals and encodes a 54-kDa protein [23]. The plasmid pHTMob02281 was used in parallel and was treated similarly to *cry6Aa*-pHTMob02281. Using the conjugative plasmid pAW63::Tn5401, *cry6Aa*-pHTMob02281 and pHTMob02281 could be transferred to all recipient *B. thuringiensis* and *B. cereus* strains with a transfer efficiency ranging from 10^−7^ to 10^−5^ transconjugants/donor (Table 1). Based on phase-contrast microscopy, all transconjugants containing *cry6Aa*-pHTMob02281 produced rice-shaped crystals, and SDS-PAGE showed that all transconjugants containing *cry6Aa*-pHTMob02281 produced parasporal crystals with a 54-kDa band representing Cry6Aa (Fig. 1).

**Table 1:** Transfer efficiency of *cry6Aa*-pHTMob02281 and pHTMob02281 in *B. thuringiensis* and *B. cereus* strains

Plasmid donors	Recipients	Efficiency	Plasmid donors	Recipients	Efficiency
	BMB171Str	4.5 × 10^−5^		BMB171Str	3.4 × 10^−5^
	YBT1520Str	8.2 × 10^−6^		YBT1520Str	9.3 × 10^−6^
*cry6Aa*-pHTMob02281	CT-43::Kan	3.0 × 10^−5^	pHTMob02281	CT-43::Kan	2.8 × 10^−5^
	UW85R	2.3 × 10^−5^		UW85R	2.7 × 10^−5^
	BC10987Str	5.3 × 10^−6^		BC10987Str	7.0 × 10^−6^

Transfer efficiency = transconjugants/donor cells

### Relaxase Mob02281 mediates the excision of the resistance marker between the 24-bp core *oriT* sites

**Fig. 2:**
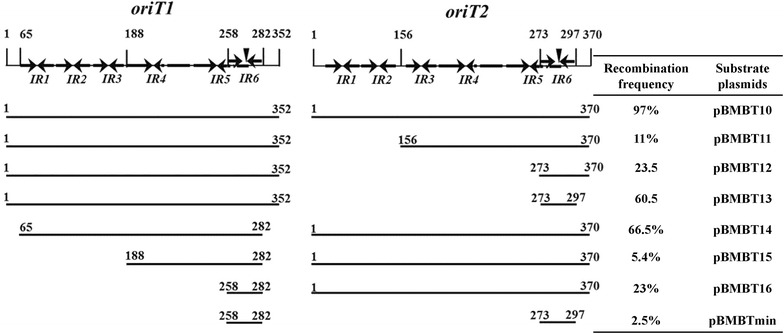
Effects of *oriT* deletions on Mob02281-mediated recombination. The 1–352 bp *oriT1* and 1–370 bp *oriT2* segments are numbered. The structures were shown in [37]. *Arrows* indicate the presence of inverted repeats (IRs). The *nic* site is indicated as a *vertical arrowhead*. The arrangements of the two *oriT* sequences with or without deletion mutations in each substrate plasmid are listed. Deleted *oriT* was cloned at the corresponding site into pBMBT10. pBMBmob1 was used as a helper plasmid containing the gene encoding a mobilization protein. The frequency of *oriT*-specific recombination for each substrate plasmid was estimated following the methods described in [37]

To decrease the influence of DNA remnants after Mob02281-mediated recombination, we identified the minimal *oriT* sequence required for Mob02281-mediated recombination. Using the previous recombination system [19], we performed a series of deletions in one or both *oriT*s (352-bp *oriT1* and 370-bp *oriT2*) of the substrate plasmid pBMBT10. The pBMBmob1 plasmid containing Mob02281 was used as a helper plasmid. As shown in Fig. 2, deletions at the two *oriT* sites had different effects on recombination activity. Compared with the high recombination frequency observed when the substrate plasmid contains bp 1–352 of *oriT1* and bp 1–370 of *oriT2*, the recombination frequency exhibited a sharp decrease when inverted repeat *IR1* and *IR2* were deleted from *oriT2*. Surprisingly, it showed a relatively higher recombination frequency when the substrate plasmid contains bp 1–352 of *oriT1* and bp 273–297 of *oriT2* having only 24 bp spanning *IR6*. However, by remaining the 370-bp *oriT2* sequence unchanged, the deletion of *oriT1* had a strong influence on recombination frequency.

**Fig. 3:**
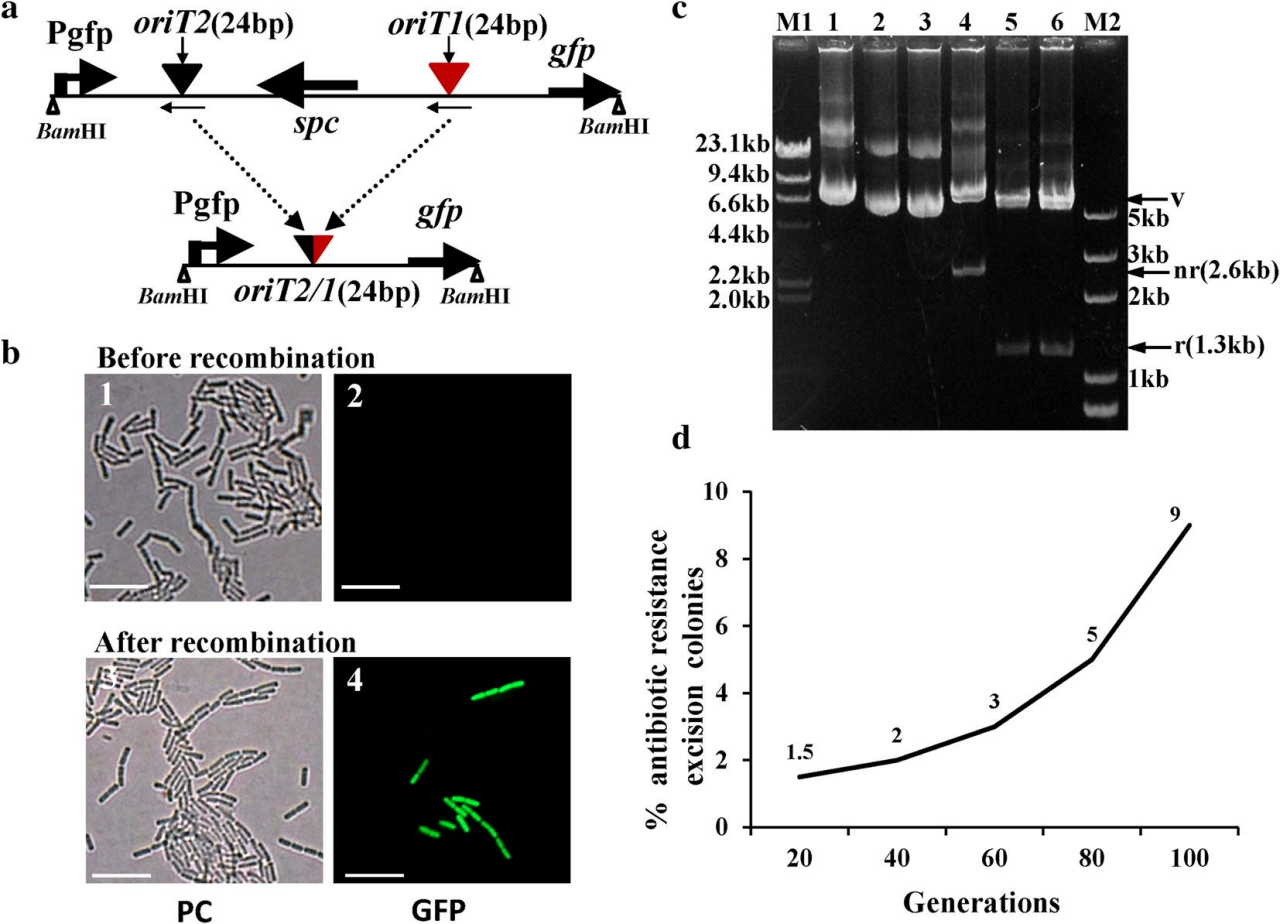
Recombination frequency of pBMBTmini. **a** Structure of the recombination cassette and the resulting product after recombination. *Arrows* indicate the direction of transcription; *spc* spectinomycin-resistance gene, *Pgfp* the promoter of the kanamycin-resistance gene, *gfp* gene encoding green fluorescent protein. **b** Observation of BMB171 (pBMBTmini + pBMBmob1) before and after recombination. *PC* phase-contrast microscopy; *GFP* fluorescence microscopy. *Bar* indicates 10 μm. **c** Restriction enzyme digestion of substrate plasmid before and after recombination; *M1* λDNA/*Hin*dIII marker; *M2* *Trans*2 K Plus DNA marker.1, before recombination, substrate plasmid pBMBTmini; 2–3, from two single Spc^S^ colonies after recombination; 4, before recombination, substrate plasmid pBMBTmini, digested with *Bam*HI; 5–6, from two single Spc^S^ colonies after recombination, digested with *Bam*HI; *v* vector, 6.5 kb; *nr* non-recombined cassette, 2.6 kb. *r* recombined cassette, 1.3 kb. **d** Increase of the percentage of antibiotic-resistance excision colonies over the number of generations

When the substrate plasmid contains bp 258–282 of *oriT1* and bp 273–297 of *oriT2* (i.e., only 24 bp of inverted repeat sequences, including the *nic* site), recombination was observed (Fig. 3). A schematic diagram of substrate construction is shown in Fig. 3a. Additionally, fluorescent BMB171 (pBMBTmini + pBMBmob1) cells were observed under phase contrast microscopy and fluorescence microscopy after recombination (Fig. 3b). Single Spc^S^ colonies were confirmed by a restriction analysis (Fig. 3c) and sequencing (Additional file 1: Fig. S1). When the cultures were grown for 20 generations, three Spc^S^ colonies out of 200 total colonies were obtained. Although this was a low level of recombination, it was sufficient to obtain a recombined plasmid. With additional generations, more Spc^S^ colonies were obtained. When the cultures were grown for 100 generations, nine Spc^S^ colonies out of 100 colonies were obtained (Fig. 3d). These results indicate that the Mob02281/mini-*oriT* (24 bp of *oriT1* and 24 bp of *oriT2*) system can mediate target gene deletion.

### Construction of a Ts mobilizable site-specific recombination vector for unmarked genetic manipulation

**Fig. 4:**
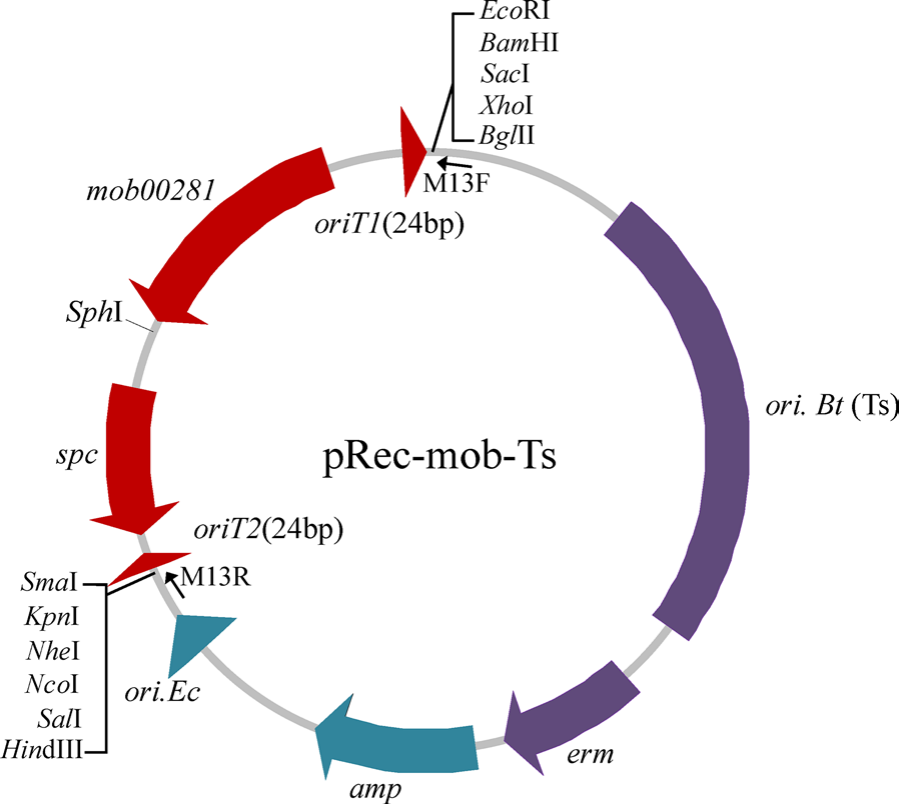
Map of the temperature-sensitive-mobilizable recombination vector pRec-mob1-Ts. *Arrows* indicate the direction of transcription or replication. The Mob-Spc cassette is shown in *red*, including the spectinomycin-resistance gene (*spc*), the *mob02281* gene, and the two mini-*oriT* sites (24-bp). The orientations of the replication of *E. coli* (*ori.Ec*) and the ampicillin-resistance gene (*amp*) are shown in *light green*; the orientations of the replication of *B. thuringiensis* and the erythromycin-resistance gene (*erm*) are shown in *purple*. The *purple box* flanking *ori.Bt (Ts*) indicates that the replication orientation of the temperature-sensitive replication region of *B. thuringiensis* is not known

We confirmed the mobilization ability of the Mob02281 module and site-specific recombination mediated by Mob02281. We then constructed a modular vector, pRec-mob1-Ts, to facilitate the use of these components for the genetic manipulation of *B. thuringiensis *and *B. cereus* strains. The crucial element of this modular plasmid is the Mob-Spc cassette, which contains the Mob02281 mobilizable module and a spectinomycin-resistance gene, flanked by the two 24-bp *oriT* core sites in the same orientation and multiple restriction sites for subcloning the homologous arms of the target gene (Fig. 4).

### Construction of an integration vector for unmarked genetic manipulation

**Fig. 5:**
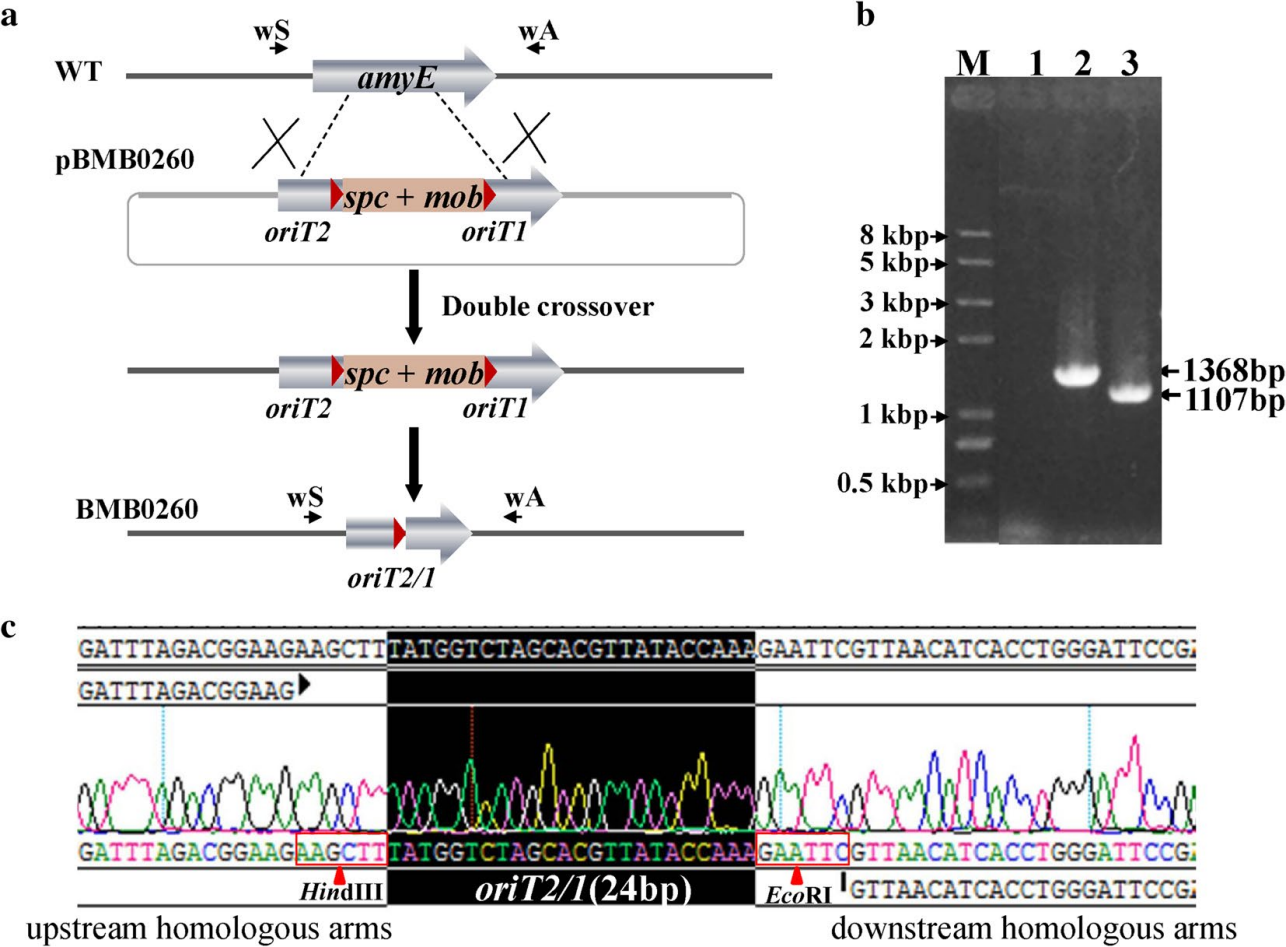
Design and confirmation of *amyE* disruption in *B. thuringiensis* BMB171. **a** Depiction of *amyE* disruption. **b** PCR detection with primers amyE-wS and amyE-wA. DNA templates were from: *1* pBMB0260 (negative control); *2* BMB171; *3* *amyE* gene-disruption strain BMB0260; *M* *Trans*2 K Plus II DNA marker; **c** Sequence analysis of the deletion region of BMB0260

To develop a universal integration vector for target gene or gene cluster expression in *B. thuringiensis* and *B. cereus* strains, a putative amylase gene was used because it is not essential and is conserved in *B. thuringiensis* and *B. cereus*. The *amyE* upstream and downstream homologous arms separated by the Mob-Spc cassette were constructed to disrupt amylase based on pRec-mob-Ts (Fig. 5a), generating the integration vector plasmid pBMB0260. Then, pBMB0260 was used to transform *B. thuringiensis* BMB171 by electroporation. BMB0260::MobSpc was obtained by using the spectinomycin-resistance gene as the selection marker. The unmarked mutant strain BMB0260 was then obtained by culturing BMB0260::MobSpc in LB medium at 28 °C; after approximately 30 generations, two spectinomycin-sensitive colonies were obtained and confirmed by PCR using primers outside the homologous arms (Fig. 5b). The PCR product was sequenced to confirm that BMB0260 was constructed by replacing a 297-bp internal *amyE* gene fragment with a 36-bp segment that contains the 24-bp *oriT* and two 6-bp restriction sites (Fig. 5c).

We tested the α-amylase activity of the *amyE* mutant strain BMB0260 using a starch-plate assay. Two single colonies of BMB0260 exhibited a circle indicating starch hydrolysis that was similar to that of BMB171, which carried an intact amylase gene (Additional file 1: Fig. S2). Based on the genome sequence analysis, we found that there are two putative amylase genes in most *B. thuringiensis* and *B. cereus* strains, such as *BMB171_C1018* encoding alpha-amylase and *BMB171_C3160* encoding cytoplasmic alpha-amylase in BMB171. The deleted *BMB171_C1018* gene was a putative alpha amylase, but its α-amylase activity has not been confirmed. This result suggests that this amylase gene has very low or no activity.

### Unmarked integration of *cry5Ba* or *cry2Aa* at the amylase locus

**Fig. 6:**
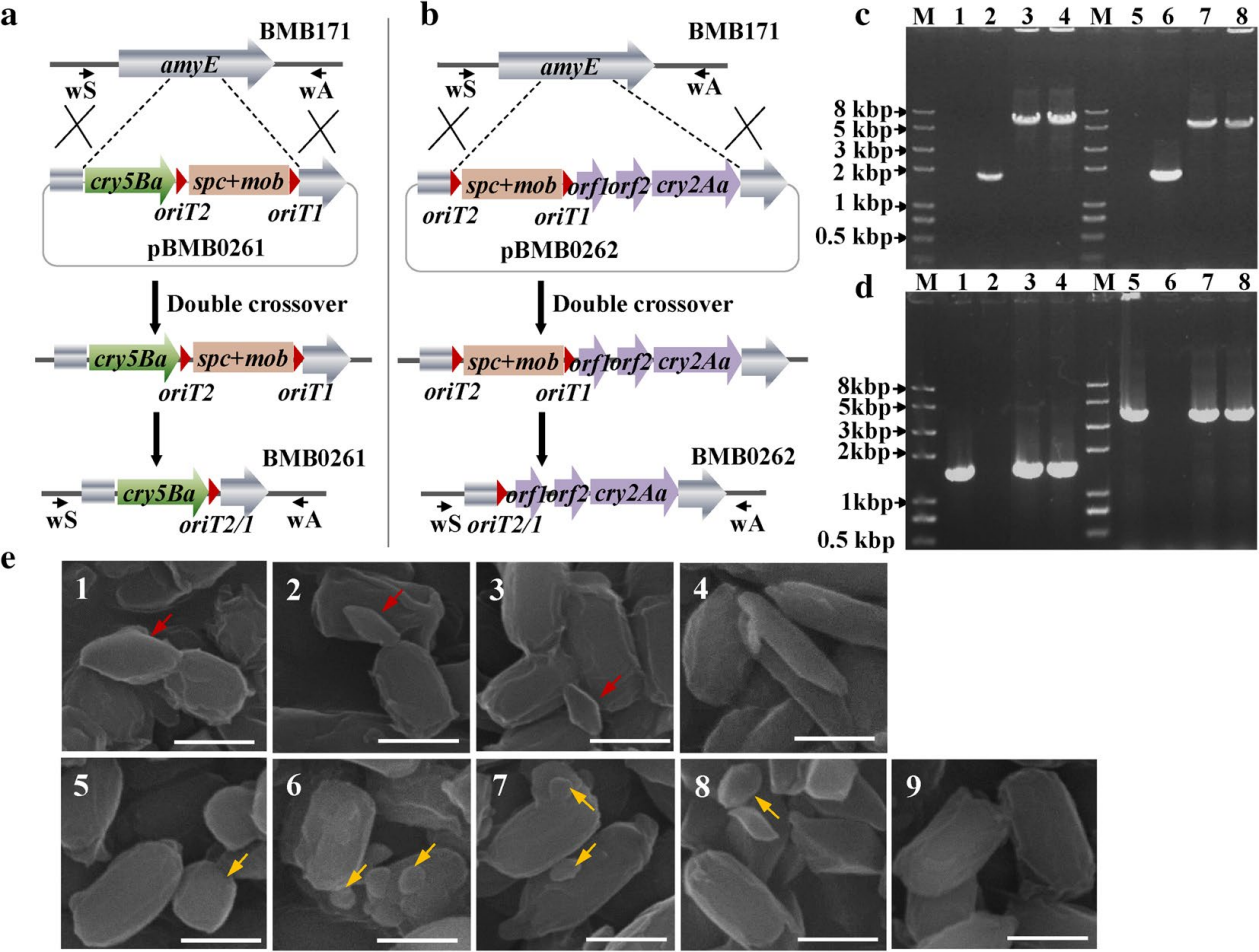
Construction of crystal protein gene insertion mutants BMB0261 and BMB0262. Depiction of *cry5Ba* (**a**) and *cry2Aa* (**b**) insertion. PCR detection with primers amyE-wS and amyE-wA (**c**) and 5B-S and 5B-A (*lanes* 1–4) or 2A-S and 2A-A (*lanes* 5–8) (**d**); DNA templates were from: *1*, pBMB0261 (negative control); *2* and *6*, BMB171; *3*–*4*, mutant BMB0261; *5*, pBMB0262 (negative control); *7*–*8*, mutant BMB0262; *M* *Trans*2 K Plus II DNA marker. **e** Scanning electron microscopy image of parasporal crystals in unmarked *cry* integrate mutants. *1*, BMB171/*cry5Ba*-pHT304; *2*–*3*, BMB0261; *4*, YBT-1518; *5*, BMB171/*cry2Aa*-pHT304; *6*–*7*, BMB0262; *8*, CT-43; *9*, BMB171. The *arrows* indicate the *diamond*-*shaped* crystals encoded by *cry5Ba* and the *round*-*shaped* crystals encoded by *cry2Aa*. *Bar* indicates 1 μm

The plasmids pBMB0261 and pBMB0262 respectively containing *cry5Ba* gene and *cry2Aa* expression cassette were used to transform *B. thuringiensis* BMB171 (Fig. 6a, b). After the Mob-spc insertion mutants were obtained, the unmarked integration mutants were generated following similar methods to those for *amyE* disruption, and a similar Mob-spc cassette excision event for the two *cry* integration mutants occurred. The excision frequencies of the MobSpc cassette in BMB0261::MobSpc (3 out of 100 colonies) and BMB0262::MobSpc (2 out of 100 colonies) were similar to that of BMB0260::MobSpc. Using PCR, we confirmed that the *cry* gene expression cassettes were integrated at the *amyE* site, together with the *oriT* fragment, in the unmarked BMB0261 and BMB0262 mutants (Fig. 6c, d). The PCR products were sequenced to confirm that BMB0261 and BMB0262 were properly constructed by integrating the 4646-bp *cry5Ba* gene and the 3939-bp *cry2Aa* expression cassette at the *amyE* locus flanking the 24-bp *oriT2*/*1* site.

Based on phase-contrast microscopy and scanning electron microscopy of the two *cry* integration mutants, the BMB0261 strains produced diamond-shaped crystals, and the BMB0262 strains produced round-shaped crystals (Fig. 6e; Additional file 1: Fig. S3A). Compared with the recombinant strains BMB171/*cry5Ba*-pHT304 and BMB171/*cry2Aa*-pHT304, in which the *cry* genes were cloned into the pHT304 vector, and the resulting plasmids were used to transform BMB171, the crystals produced by BMB0261 and BMB0262 were much smaller. This may be because the copy number of the plasmid vector pHT304 is much higher than a one-copy chromosome. Based on SDS-PAGE, protein expression is lower in BMB0261 and BMB0262 than the recombinant stains BMB171/*cry5Ba*-pHT304 and BMB171/*cry2Aa*-pHT304, which supports this interpretation (Additional file 1: Fig. S3B). The development of the integration strains BMB0261 and BMB0262, results in bacterial production of insecticidal crystal proteins, especially for *cry5Ba*, which is silent in its original strain YBT-1518.

### Unmarked disruption of a seryl-AMP synthetase gene in zwittermicin A biosynthetic gene cluster

**Fig. 7:**
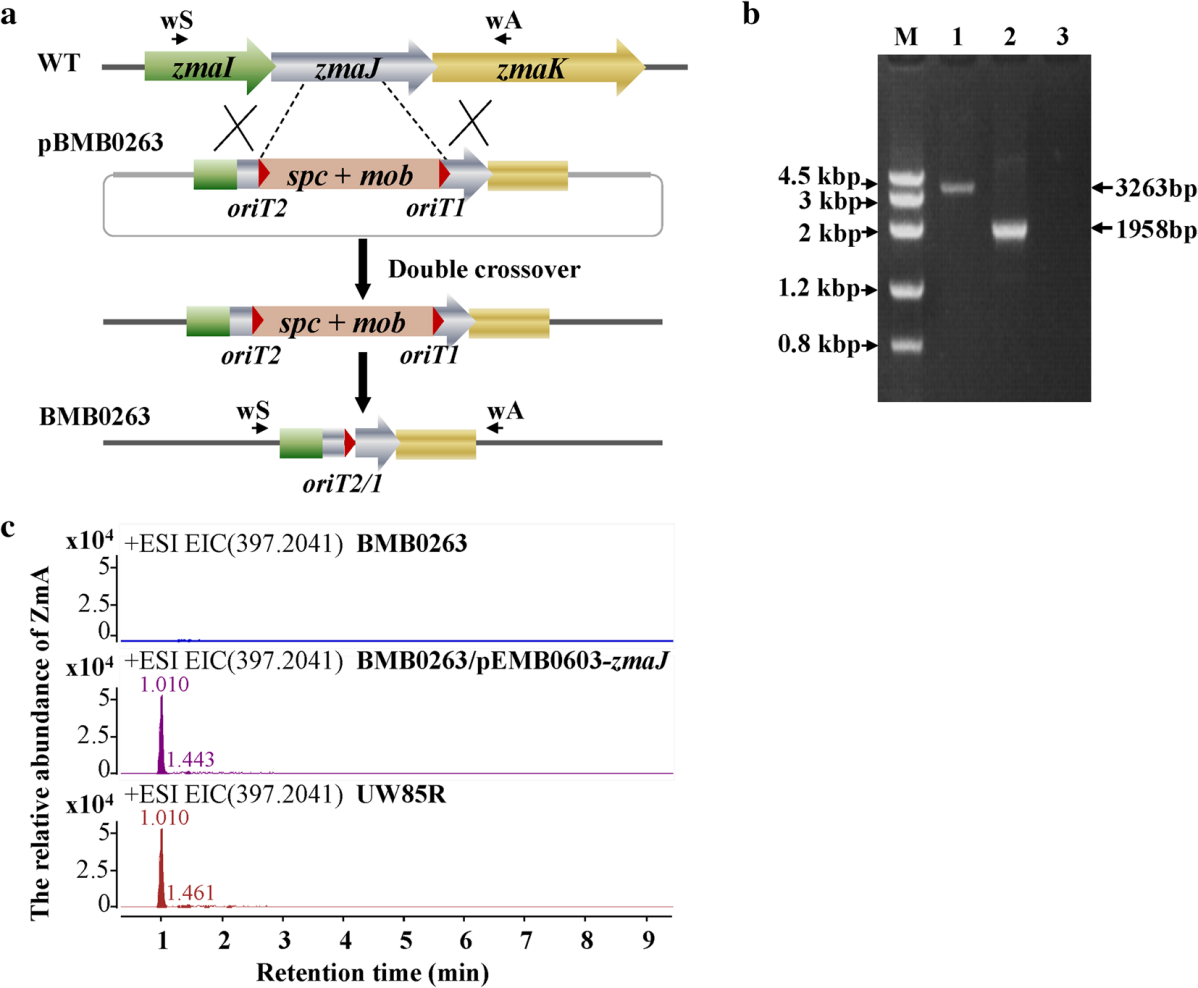
Design and confirmation of *zmaJ* disruption in *B. cereus* UW85R. **a** Depiction of *zmaJ* disruption. **b** PCR detection with primers zmaJ-wS and zmaJ-wA. DNA templates were from: *1*, UW85R; *2*, *zmaJ* gene-disrupted strain BMB0263; *3*, pBMB0263 (negative control); *M*, DNA Marker III. **c** LC–MS detection of a molecular weight corresponding to ZmA in the culture supernatants. The molecular weight corresponding to ZmA is indicated by a *rectangular frame*

To test whether this Mob/*oriT* recombination system could be used for the unmarked disruption in *B. cereus*, the *zmaJ* gene, which encodes seryl-AMP synthetase involved in (*2S*) amino malonyl-ACP formation in the zwittermicin A (ZmA) biosynthetic gene cluster [24] was selected as a deletion target. Plasmid pBMB0263 was constructed to disrupt *zmaJ* using pRec-mob-Ts (Fig. 7a). The unmarked integration mutants were generated following similar methods to those for *amyE* disruption and *cry* gene insertion. The unmarked integration mutants were confirmed by PCR using primers outside the homologous arms (Fig. 7b). The excision frequencies of the MobSpc cassette in BMB0263::MobSpc (3 out of 100 colonies) were similar to that of BMB0260::MobSpc. The PCR product was sequenced to confirm that BMB0263 was constructed by replacing a 1341-bp internal *zmaJ* gene fragment with a 36-bp segment containing the 24-bp *oriT* and two 6-bp restriction sites. As shown in Fig. 7c, using high resolution LC–MS to detect the production of ZmA, the *zmaJ* deletion mutant BMB0263 cannot produce ZmA, unlike UW85R. Thus, gene deletion via Mob-mediated recombination is feasible in *B. cereus*. In addition, complementation of *zmaJ* via pEMB0603-*zmaJ* to BMB0263 rescued the wild-type phenotype.

## Discussion

Several *B. thuringiensis *and *B. cereus* strains have low electroporation efficiency or are not competent for transformation [25, 26], thus rendering DNA transfer by conjugation is the method of choice. To test the mobilization ability of Mob02281 into various *B. thuringiensis* and *B. cereus* strains, the previously constructed mobilizable plasmid pHTMob02281, which contains Mob02281 with *oriT1*, was used [19]. The successful transfer of pHTMob02281 into various *B. thuringiensis* and *B. cereus* strains was confirmed. Thus, pHTMob02281 can be used as a mobilizable shuttle vector for gene expression. We also confirmed its mobilization ability during the construction of an in-frame *zmaJ* deletion mutant in *B. cereus* UW85R. Therefore, this Mob02281/*oriT* system provides an alternative approach for genetic manipulations in these strains, which have rather low electroporation efficiencies.

Using this approach, the spectinomycin-resistant gene can be used to screen the Mob-Spc cassette integration mutants at the target locus. Antibiotics can be added to the medium to ensure that the excision of the cassette does not occur prematurely. Increasing the number of generations can increase the recombination frequency, and the excision of the Mob-Spc cassette occurs at the optimum growth temperature for *B. thuringiensis* and *B. cereus* strains.

We temporarily inserted an antibiotic resistance cassette flanked by pBMB0228 *oriT* sites oriented as direct repeats into a specific genomic region and subsequently removed it using the Mob/*oriT* system. The absence of the antibiotic resistance markers assures that cell physiology is not altered by known or unrecognized activities of resistance genes or antibiotic drugs. The *cry5Ba* gene is a nematicidal protein gene, but it is silent in its original strain YBT-1518 [23], and *cry2Aa* cassette encodes proteins that form insecticidal crystal proteins active against lepidopteran and dipteran larvae [27]. Using this unmarked system, we constructed *cry5Ba* and *cry2Aa* integration strains in *B. thuringiensis* BMB171. The two strains with *cry* integration can be used as genetically modify bacteria for the production the insecticidal crystal proteins because they avoid contamination and the risk of antibiotic resistance gene transfer to the environment. Although the protein expression is lower in the two strains with *cry* integration than the recombinant strains containing the *cry* genes maintained in a plasmid vector because the copy number of a one-copy chromosome is much less than the plasmid vector, the protein production can be improved by replacing the original promoter with a strong promoter to express the *cry* genes. In the long term, the *cry* gene integrated in the chromosome is much stable than maintained in a plasmid vector. Thus, Mob/*oriT* system provides an efficient marker-free approach for constructing genetically modify bacteria.

The *zmaJ* gene within the operon of the ZmA biosynthetic gene cluster was selected as a target for deletion. Complementation of *zmaJ* to the *zmaJ* deletion strain rescued the wild-type phenotype. This result suggests that the 36-bp DNA remnants after Mob02281-mediated recombination did not affect the expression of other genes in the operon.

In all current site-specific recombination systems, including the FLP/*FRT* system [6–8], Cre/*loxP* system [9, 10], Xer/*dif* system [11, 12], and Relaxase*/oriT* system [16, 28], a single recombination site remains within the target region, and undesired recombination might occur between recombination sites that accumulate after multiple rounds of gene disruption. In the Cre/*loxP* system, Cre recombinase activity was limited to produce defined recombination events in strains containing multiple *loxP* sites [10]. The combination of these systems may resolve these issues when multiple genes are manipulated in a target strain. In this study, to minimize undesired recombination using the Mob02281/*oriT* system, we used the original promoter, rather than an inducible strong promoter, to express Mob02281.

A TnpI-mediated recombination system has been used to construct genetically engineered *B. thuringiensis* strains. The *cry1C* gene is introduced and maintained in a plasmid vector whose antibiotic resistance genes are flanked by two internal resolution sites. The antibiotic resistance genes of the plasmid are then eliminated by TnpI-mediated recombination between the two internal resolution sites [29]. However, the long-term stability of *cry1C* expression is an issue. Moreover, this method has not been assessed with respect to gene deletion. In *B. anthracis*, the Cre/*loxP* system can be used to efficiently inactivate individual genes and facilitate large deletions [10]. In this approach, two temperature-sensitive plasmids are electroporated into the target strain. One contains a resistance cassette flanked by *loxP* sites and is used to temporarily insert the resistance gene into a selected genomic region based on homologous sequences. The other expresses the Cre recombinase and is used to remove the resistance gene after recombination between *loxP* sites [10]. This process cannot be applied to some wild-type *B. cereus* group strains in which electroporation are inefficient.

We also tested the recombination ability of the Mob02281/*oriT* system in other bacterial taxa; its inefficiency in *E. coli* indicated that it might have specific host requirements. However, in addition to Mob02281, certain relaxases can catalyze site-specific recombination between two *oriT* copies, including NikB of plasmid R64 [16], TrwC of plasmid R388 [17], MobA of plasmid R1162 [18] from Gram-negative bacteria, MobB and MobE of plasmid pAMα1 [30], and the Mobs of plasmid pE194 and pT181 [31] from Gram-positive bacteria. Therefore, future studies should examine various relaxase/*oriT* elements to extend the applications of this alternative recombination system for unmarked genetic manipulations to a wider range of taxa, including both Gram-negative and Gram-positive bacteria.

## Conclusions

The Mob/*oriT* mobilizable recombination system can be exploited for unmarked genetic manipulation and construction of genetically modify bacteria in *B. thuringiensis* and *B. cereus*. Using this system, the recombination vectors were constructed and the application was tested by in-frame deletion in *B. thuringiensis* and *B. cereus* and insertion the interested gene into chromosome in *B. thuringiensis.* We also confirmed the putative amylase gene can be used as integration site for gene expression in *B. thuringiensis* and *B. cereus*.

## Methods

### Bacterial strains, plasmids, growth conditions, and DNA manipulations

The strains and plasmids used in this study and their sources are listed in Table 2. *E. coli* was cultured in LB medium at 37 °C, and all *B. thuringiensis* and *B. cereus* strains were cultured in LB medium at 28 °C. Spectinomycin, tetracycline, erythromycin, kanamycin, ampicillin, rifampin, and streptomycin were added to the media at final concentrations of 100, 10, 25, 50, 100, 100, and 100 μg/mL, respectively. All regular DNA manipulations were carried out following standard methods [32]. All recombinant plasmids were constructed in *E. coli*, and plasmids were extracted from *E. coli* according to the methods of Sambrook and Russell [32]. PCR products were confirmed by DNA sequencing. Primers used for PCR amplification are listed in Table 3.

**Table 2:** Bacterial strains and plasmids used in this study

Strains or plasmids	Description^a^	Reference
*Bacillus thuringiensis* strains		
AW48	Derivative of wild-type isolate HD73 cured of pHT73, containing pAW63::Tn5401, Tet^r^	[36]
BMB0260	Mutant of strain BMB171 in which the *amyE* gene is interrupted by in-frame deletion	This study
BMB0261	Mutant of strain BMB171 in which the *cry5Ba* gene is inserted in the *amyE* locus	This study
BMB0262	Mutant of strain BMB171 in which the *cry2Aa* expression cassette is inserted in the *amyE* locus	This study
BMB171	Acrystalliferous mutant of *B. thuringiensis*	[39]
BMB171Str	Streptomycin spontaneous mutant strain of BMB171, Str^r^	This study
YBT-1520Str	Streptomycin spontaneous mutant strain of *B. thuringiensis* serovar *kurstaki* YBT-1520, Str^r^	This study
CT-43	No flagellum, containing *cry2Aa* expression cassette and thuringiensin synthesis cluster	[40]
CT-43::Kan	Mutant of strain CT-43 in which the *thuE* gene is interrupted by the kanamycin-coding gene, also named BMB0545, Kan^r^	[41]
*Bacillus cereus* strains		
ATCC 10987Str	Streptomycin spontaneous mutant strain of *B. cereus* ATCC 10987, Str^r^	This study
UW85	Wild type *B. cereus* strain, ZmA producing	[24]
UW85R	Rifampin spontaneous mutant resistance strain of *B. cereus* UW85, Rif^r^	This study
BMB0263	Mutant of strain UW85R in which the *zmaJ* gene is interrupted by in-frame deletion	This study
*Escherichia coli* strains		
DH5α	*F*-*φ80lacZΔM15 Δ(lacZYA*-*argF) U169 deoR recA1 endA1 hsdR17 (rk*-*,mk*+*) phoA supE44 λ*- *thi*-*1 gyrA96 relA1*	Invitrogen
Plasmids		
pAW63::Tn5401	A self-transmissible plasmid generated by insertion of the tetracycline-resistance transposon Tn5401 in pAW63, Tet^r^	[36]
pBMB0215	A recombinant plasmid harboring a 4.6-kb *Hin*dIII fragment including *cry5Ba*	[23]
pBMB0228	17,706-bp endogenous plasmid in strain YBT-1518	[19, 42]
pBMBmob1	pEMB0603 containing a 1.8-kb *Bam*HI/*Sph*I fragment carrying *mob02281*	[19]
pBMBT10	pHT315 containing the recombination cassette with bp 1–352 of *oriT*1 and bp 1–370 of *oriT*2	[19]
pBMBT11	pHT315 containing the recombination cassette with bp 1–352 of *oriT*1 and bp 156–370 of *oriT*2	This study
pBMBT12	pHT315 containing the recombination cassette with bp 1–352 of *oriT*1 and bp 273–370 of *oriT*2	This study
pBMBT13	pHT315 containing the recombination cassette with bp 1–352 of *oriT*1 and bp 273–297 of *oriT*2	This study
pBMBT14	pHT315 containing the recombination cassette with bp 65–282 of *oriT*1 and bp 1–370 of *oriT*2	This study
pBMBT15	pHT315 containing the recombination cassette with bp 188–282 of *oriT*1 and bp 1–370 of *oriT*2	This study
pBMBT16	pHT315 containing the recombination cassette with bp 258–282 of *oriT*1 and bp 1–370 of *oriT*2	This study
pBMBTmini	pHT315 containing the recombination cassette as in pBMBT10 [19] with the 24-bp core sequence of *oriT*1 and 24-bp core sequence of *oriT*2	This study
pEMB0603	*E. coli* and *B. thuringiensis* shuttle vector, Amp^r^, Kan^r^	[35]
pEMB0603-*zmaJ*	pEMB0603 containing a 2.1 kb *Eco*RI/*Sal*I fragment carrying the coding sequence of the seryl-AMP synthetase gene *ZmaJ* with the promoter of kanamycin resistance gene	This study
pHT315	*E. coli* and *B. thuringiensis* shuttle vector, Amp^r^, Erm^r^	[43]
pHT304	*E. coli* and *B. thuringiensis* shuttle vector, Amp^r^, Erm^r^	[43]
pHT304Ts	*E. coli* and *B. thuringiensis* shuttle vector, containing a temperature-sensitive replicon in *B. thuringiensis*, Amp^r^, Erm^r^	[35]
pHTMob02281	pHT304 containing a 1.8-kb *Hin*dIII fragment carrying *mob02281* and *oriT*1	This study
pIC333	An efficient mini-Tnl0 delivery vector in *B. subtilis*, Spc^r^, Erm^r^	[33]
pRec-mob1-Ts	pHT304Ts containing the Mob-Spc cassette and 24-bp core *oriT* recombination sequence, Amp^r^, Erm^r^, Spc^r^	This study

^a^ *Amp* ^*r*^ ampicillin resistance, *Erm* ^*r*^ erythromycin resistance, *Kan* ^*r*^ kanamycin resistance, *Tet* ^*r*^ tetracycline resistance, *Spc* ^*r*^ spectinomycin resistance, *Str* ^*r*^ streptomycin resistance, *Rif* ^*r*^ rifampin resistance

**Table 3:** Primers used in this study

Primers	Sequence^a^ (5′–3′)	Construct(s) or use
oriT1-1S	CGA*GAATTC*GTAAGCGGAACGAAGTG	PCR amplification of bp 1–352, 65–282 and 188–282 of *oriT1*
oriT1-65S	CGA*GAATTC*TGTTAAAACGGTTACCGTTTTG	
oriT1-352A	CAG*GTCGAC*CTACATGAGCCACGATCAATC	
oriT1-282A	CAG*GTCGAC*TTTGGTCTAGCACGTTATACC	
oriT1-188A	CAG*GTCGAC*GACTTTGTCCGAACCTACAAG	
oriT2-1S	TAT*GCATGC*CAAAGGTACCGGACCGAACC	PCR amplification of bp 1–370, 156–370 and 273–370 of *oriT2*
oriT2-370A	CAG*AAGCTT*GAACGTGAGCCATGAGCGAG	
oriT2-156S	TAT*GCATGC*GAAGGGGGTCAAGGGGAATT	
oriT2-273S	TAT*GCATGC*ATTTGGTATAACGTGCTAGAC	
oriT1-24-S	TAT*GAATTCT*TTGGTATAACGTGCTAGACCAAATCTGACGCTCAGTGG	pBMBT13, pBMBT16 and pBMBTmini
oriT2-24-A	CCC*AAGCTT*TATGGTCTAGCACGTTATACCAAAAAATGTCACTAATATT	
MS	*GAATTCGGATCCGAGCTCCTCGAGAGATCT*TTTGGTCTAGCACGTTATACCAAAGTGTGTTCTTAATATTGTATG	pRec-mob1-Ts
MA	CGC*GTCGACGCATGC*GATTACAACAAGTCTATTCAG	
SS	CGC*GCATGC*TCTGACGCTCAGTGGAAC	
SA	*GTCGACCCATGGGCTAGCGGTACCCGGG*TTTGGTATAACGTGCTAGACCATACTATGCAAGGAACAATTTC	
amyE-up-S	CGC*GAATTC*ATGCGTTTCTCTCGGATAATTGG	BMB0260, BMB0261 and BMB0262
amyE-up-A	CTC*GGATCC*CTTCCGTCTAAATCAACTTC	
amyE-down-S	CGC*GTCGAC*GTTAACATCACCTGGGATTC	
amyE-down-A	GCC*AAGCTT*TCATCTACGCTTCCTTTTAACTG	
amyE-wS	GTGCCAATCCTGGTGTCATC	
amyE-wA	GAAAATCCTCTTCCTAACCTG	
5B-S	GGGTATACAAGAAGGTTGGG	*cry5Ba* detection
5B-A	CGTTTTCTGGTACAAGTTCC	
2A-S	GCC*GAATTC*GATGTTGATTCTTAGAGCAATG	BMB0262
2A-A	GGC*GAATTC*GGTTAACTTGAAATGATTTCTCC	
zmaJ-up-S	CGG*GAATTC*CATTGGTAGACCAGGATTTGC	BMB0263
zmaJ-up-A	CAT*GGATCC*TCCACCTTCACGTAAAAGACG	
zmaJ-down-S	CGG*GGTACC*GTACTTGCCTTATTAGGTTCCG	
zmaJ-down-A	CAC*GTCGAC*ACTTACACGAAGTGGTGGTG	
zmaJ-wS	CAGCTGCTGATGTGTTTTTGG	
zmaJ-wA	CTCAACTACCATAATAGTGTC	
pKan-S	CCG*GAATTC*CATTTGAGGTGATAGGTAAG	pEMB0603-*zmaJ*
pKan-zmaJ-A	TATACATGTACTCATTTTAATAACCTCCTTTCTCTAGACCCCAAGAAGCTAATTATAAC	
pKan-zmaJ-S	ATTAGCTTCTTGGGGTCTAGAGAAAGGAGGTTATTAAAATGAGTACATGTATACAAAAG	
zmaJ-A	ACGC*GTCGAC*TTATGAATCTTTACTGCTTTC	

^a^Restriction sites included in oligonucleotide sequences are italicized

### Transformation of *E. coli* and *B. thuringiensis*

*E. coli* transformation was carried out using CaCl_2_-treated competent cells, as described by Sambrook [32]. *B. thuringiensis* transformation was performed by electroporation with the Bio-Rad Gene Pulser (Hercules, CA, USA) with previously described settings [25].

### Plasmid construction

To decrease the influence of DNA remnants after Mob02281-mediated recombination, we identified the minimal *oriT* sequence required for Mob02281-mediated recombination. Using the previous recombination system [19], we performed a series of deletions in one or both *oriT*s (352-bp *oriT1* and 370-bp *oriT2*) of the substrate plasmid pBMBT10.

The substrate plasmids pBMBT11, pBMBT12, pBMBT13, pBMBT14, pBMBT15, pBMBT16 and pBMBTmini was constructed with a similar organization to that of pBMBT10 [19]. The truncated *oriT1* fragments containing bp 1–352, 65–282 and 188–282 were respectively amplified from pBMB0228 using the primer pair oriT1-1S/oriT1-352A, oriT1-65S/oriT1-282A, oriT1-188S/oriT1-282A. The truncated *oriT2* fragments containing bp 1–370, 156–370 and 273–370 were respectively amplified from pBMB0228 using the primer pair oriT2-1S/oriT2-370A, oriT2-156S/oriT2-370A and oriT2-273S/oriT2-370A. Plasmids pBMBT11, pBMBT12 and pBMBT13 were constructed by remaining the bp 1–352 *oriT1* unchanged, and respectively containing the bp 156–370, 273–370, and 273–297 of *oriT2*. Plasmids pBMBT14, pBMBT15 and pBMBT16 were constructed by remaining the bp 1–370 of *oriT2* unchanged, and respectively containing the bp 65–282, 188–282 and 258-282 of *oriT1*. Plasmid pBMBTmini contains 24-bp core *oriT1* and 24-bp core *oriT2* sequences. The two 24-bp core *oriT* sequences in the same orientation flanking a spectinomycin-resistance marker were amplified with the primer pair oriT1-24-S/oriT1-24-A using pBMBT10 as a template. Other construction steps of these substrate plasmids were similar to those described previously for the construction of pBMBT10 [19].

The mobilizable module, which comprised the *mob02281* gene and a 352-bp *oriT1* region overlapping its promoter, was amplified from pBMB0228 using the primer pair MS/MA, and the spectinomycin-resistance gene (a selection marker) was amplified from pIC333 [33] using the primer pair SS/SA. The 1.9- and 1.3-kb PCR products were digested with *Eco*RI/*Sph*I and *Sph*I/*Sal*I, respectively, and cloned into the *Eco*RI/*Sal*I sites of the Ts vector pHT304Ts, generating the Ts mobilizable site-specific recombination vector pRec-mob-Ts. Multiple restriction sites were introduced into pRec-mob-Ts with primers by PCR amplification.

To generate the complementation plasmid pEMB0603-*zmaJ*, the promoter of kanamycin resistance gene was amplified from pBMBT10 using the primer pair pKan-S/pKan-zmaJ-A, and the coding region of the *zmaJ* gene was amplified from *B. cereus* UW85 genomic DNA using the primer pair pKan-zmaJ-S/zmaJ-A. Using overlap PCR with the primer pair pKan-S/zmaJ-A, a PCR fragment containing the coding region of *zmaJ* and a promoter of the kanamycin resistance gene was generated. Then, this 2.1-kb PCR fragment was digested with *Eco*RI/*Sal*I and inserted into the same sites of the shuttle vector pEMB0603, resulting in plasmid pEMB0603-*zmaJ*.

### Construction of unmarked mutant strains BMB0260, BMB0261, BMB0262, and BMB0263

To generate the *amyE* mutant strain BMB0260, two primer pairs, amyE-up-S/amyE-up-A and amyE-down-S/amyE-down-A, were used to amplify the upstream and downstream homologous arms of the *amyE* gene from BMB171 genomic DNA. The 566- and 529-bp fragments were digested with *Eco*RI/*Bam*HI and *Sal*I/*Hin*dIII, respectively, and cloned into the corresponding sites of pRec-mob-Ts one by one, generating pBMB0260.

To demonstrate that the Mob/*oriT* recombination system could be used to introduce a gene of interest, plasmids pBMB0261 and pBMB0262, containing *cry5Ba* and *cry2Aa* (together referred to as *cry* genes), were constructed based on the integrative vector pBMB0260. The *cry5Ba* gene was digested with *Hin*dIII from its plasmid pBMB0215 [23] and inserted into the *Hin*dIII site of pBMB0260. The *cry2Aa* expression cassette containing *orf1*, *orf2* and *cry2Aa* [34] was amplified using the primer pair 2A-S/2A-A from *B. thuringiensis* CT-43, digested with *Eco*RI, and inserted into the *Eco*RI site of pBMB0260. The resulting plasmids pBMB0261 and pBMB0262 contains *cry* genes and could be integrated into the *amyE* locus via the *amyE* homologous arms (Fig. 6a, b).

To generate the *zmaJ* mutant strain BMB0263, two primer pairs, zmaJ-up-S/zmaJ-up-A and zmaJ-down-S/zmaJ-down-A, were used to amplify the upstream and downstream homologous arms of the *zmaJ* gene from *B. cereus* UW85 genomic DNA. The 675- and 940-bp fragments were digested with *Eco*RI/*Bam*HI and *Kpn*I/*Sal*I, respectively, and cloned into the corresponding sites of pRec-mob-Ts one by one, generating pBMB0263 (Fig. 7a).

Using spectinomycin, mutants containing the Mob-Spc cassette were selected following a general method used for *B. thuringiensis* gene knockout [35]. Briefly, the Ts plasmids pBMB0260, pBMB0261, pBMB0262, and pBMB0263 were used to transform *B. thuringiensis* BMB171 by electroporation. The transformant BMB171/pBMB0263 was used as the donor strain, AW48 carrying the conjugative plasmid pAW63::Tn5401 was used as the helper strain, and *B. cereus* UW85R was used as the recipient strain. Triparental mating yielded the transconjugant UW85R/pBMB0263. The correct colonies BMB171/pBMB0260, BMB171/pBMB0261, BMB171/pBMB0262, and *B. cereus* UW85R/pBMB0263 were cultivated in LB medium containing spectinomycin (40 µg/mL) at 28 °C for 8 h. These strains were incubated at 42 °C for 4 days to generate the Mob-Spc cassette integrated mutants and eliminate unintegrated Ts plasmids.

The expected mutant strains, which were resistant to spectinomycin (100 μg/mL), but sensitive to erythromycin (25 μg/mL), were collected and confirmed by PCR using appropriate primers and sequencing. Finally, the mutant strains BMB0260::Mob-Spc, BMB0261::Mob-Spc, BMB0262::Mob-Spc and BMB0263::Mob-Spc were obtained.

The mutant strains with Mob-Spc cassette integration were cultivated at 28 °C for 24 h to excise the Mob-Spc cassette via recombination mediated by Mob02281, which is encoded by the Mob-Spc cassette. To increase the population of mutant strains from which the Mob-Spc cassette was excised, bacterial suspensions were diluted 1000-fold in fresh LB liquid medium every 8 h. The expected unmarked mutant strains, which were sensitive to spectinomycin (100 μg/mL), were collected and confirmed by PCR using primers outside the homologous arms and sequenced. Finally, the expected mutant strains BMB0260, BMB0261, BMB0262, and BMB0263 were obtained.

### Plasmid transfer

To test the mobilization ability of Mob02281 into various *B. thuringiensis* and *B. cereus* group strains, the previously constructed mobilizable plasmid pHTMob02281, which contains Mob02281 with *oriT1*, was used [19]. To facilitate phenotypic observations of DNA that is transferred to the recipient strains, the plasmid *cry6Aa*-pHTMob02281, which contains *cry6Aa* as a reporter, was constructed and used to transform *B. thuringiensis* BMB171. As recipient strains, we used *B. thuringiensis* BMB171Str, *B. thuringiensis* CT-43::Kan, and *B. thuringiensis* serovar *kurstaki* YBT-1520Str, as well as the closely related species *B.cereus* strains UW85R and ATCC 10987Str. It should be noted that *B. thuringiensis* CT-43::Kan and YBT-1520Str have rather low electroporation efficiencies [25].

Conjugation assays were conducted following the protocols described by Andrup et al. [36]. In brief, overnight cultures of donor, recipient, and helper strains were incubated at 28 °C in LB medium with appropriate antibiotics. Equal quantities of donor, recipient, and helper cells in logarithmic growth were mixed and incubated in 7 mL of pre-warmed LB medium, without antibiotics, at 28 °C with moderate shaking (180 rpm). After mating proceeded for 3 h, appropriate dilutions were plated on selective medium to determine the number of transconjugants. Control donors, recipients, and helper strains were grown separately and tested in parallel. Transfer frequencies were calculated as the ratio of transconjugants to donor cells. Each experiment was performed in triplicate.

### Recombination assays

Recombination assays were conducted following previously described methods [19]. To test the efficiency of Mob02281-mediated recombination, the substrate plasmids pBMBT11, pBMBT12, pBMBT13, pBMBT14, pBMBT15, pBMBT16 and  pBMBTmini was respectively introduced to BMB171 containing the helper plasmid pBMBmob1. The resulting colonies were grown with erythromycin and kanamycin and plated on erythromycin and kanamycin plates by dilution, and the resulting single colonies were transferred onto spectinomycin-containing plates after 20, 40, 60, 80, and 100 generations. Every 8 h (approximately 10 generations) [29], bacterial suspensions were diluted 1000-fold in 10 mL of fresh LB liquid medium. Recombination frequency was estimated as the number of spectinomycin-sensitive colonies. Each experiment was performed in triplicate.

### Microscopic observations

For observations of the number of fluorescent cells under phase-contrast and fluorescence microscopy, all *B. thuringiensis* strains were cultivated in LB liquid medium at 28°C. The morphology of the strains was observed with an Olympus photomicroscope (Tokyo, Japan).

To observe the production of parasporal crystals under phase-contrast microscopy, all strains were sporulated using a previously described method [37]. Briefly, strains were cultured in ICPM liquid medium (0.6 % peptone, 0.5 % glucose, 0.1 % CaCO_3_, 0.05 % MgSO_4_, and 0.05 % KH_2_PO_4_ (pH 7.0)) for 36 h at 28°C. The spores and crystals were collected and washed three times with 1 M NaCl solution and three times with water [37]. These samples were also used to detect crystal protein expression by sodium dodecyl sulfate polyacrylamide gel electrophoresis (SDS-PAGE). Under phase-contrast microscopy, spores are phase-bright, and crystals are phase-dark [35]. For scanning electron microscopy with a Quanta200 (FEI, Hillsboro, OR, USA), the lysed cell samples were treated following the methods described by Shao et al. [37].

### Detection of zwittermicin A (ZmA) by liquid chromatography mass spectrometry

High-resolution liquid chromatography mass spectrometry (LC–MS) was performed using an Agilent 1260 LC device (Agilent, USA) equipped with a C_18_ reverse-phase column (100 × 1.8 mm; particle size, 3.5 μm). The mobile phase consisted of chromatography grade water containing 0.1 % formic acid in pump B and chromatography grade acetonitrile in pump A. The 1uL of 36-h culture supernatants of UW85R and ZmA mutants were loaded. The elution step was performed under an acetonitrile concentration of 5 % for 5 min at a flow rate of 0.3 mL/min. The diode array detection was performed at 210 nm. MS was performed using the Q-TOF(quadrupole-time of flight) MS G6540A system (Agilent) equipped with a dual-source electrospray ionization (ESI) ion source operated in positive-ion mode. Calibration was performed with standard references of mass 121.0509 and 922.0098. The source parameters were as follows: gas temperature of 350 °C, gas flow rate of 8 L/min, and nebulizer stress of 35 lb/in^2^ (gauge). The capillary, fragmenter, skimmer, and octopole radio frequency (RF) peak voltage were set at 4000, 150, 65, and 750 V, respectively, for the scan source. The quadrupole was set to pass ions from *m/z* 100 to 1000. The MS scan rate was set to two spectra/s. Data were analyzed using Agilent MassHunter qualitative analysis software. Based on the molecular formula C_13_H_28_N_6_O_8_ of ZmA, the theoretical *m/z* of [M + H]^+^ 397.2046 (±20 ppm) was extracted to detect ZmA in each sample [24, 38].

## Additional file

10.1186/s12934-016-0492-9 Sequence analysis of the 24bp *oriT* core regions before (A) and after recombination (B). **Figure S2.** A test of the α-amylase activity of amyE mutant strain BMB0260. (A) and (B), Strain BMB171 and BMB0260 were grown on LB plates containing 1% starch; One plate (B) was stained with iodine to detect the α-amylase activity, which was indicated by the Hydrolysis circle. 1, BMB171; 2-3, BMB0260. **Figure S3**. Cry5Ba and Cry2Aa expression in the unmarked *cry* gene integration mutants of BMB171. (A). Phase contrast microscopy graph of parasporal crystals in the unmarked *cry* gene integrate mutants. The arrows respectively indicates the diamond-shaped crystals encoded by *cry5Ba* and the round-shaped crystals encoded by *cry2Aa*. (B). SDS-PAGE analysis of crystal proteins in the unmarked *cry* gene integration mutants of BMB171. The arrows indicate the bands produced by *cry5Ba* and *cry2Aa*, respectively. 1, BMB171/*cry5Ba*-pHT304; 2-3, BMB0261; 4, YBT-1518; 5, BMB171/*cry2Aa*-pHT304; 6-7, BMB0262; 8, CT-43; 9, BMB171. M, Protein ladder.
