# Supplementary material for: Mob/oriT, a mobilizable site-specific recombination system for unmarked genetic manipulation in Bacillus thuringiensis and Bacillus cereus
Source: Microb Cell Fact. 2016 Jun 10;15:108. doi: 10.1186/s12934-016-0492-9 (PMC4902927; doi:10.1186/s12934-016-0492-9)

**SUPPLEMENTARY FILE**

# Mob/*oriT*, a mobilizable site-specific recombination system for unmarked genetic manipulation in *Bacillus thuringiensis* and *Bacillus cereus*

Pengxia Wang**^†^**, Yiguang Zhu**^†^**, Yuyang Zhang, Chunyi Zhang, Jianyi Xu, Yun Deng, Donghai Peng, Lifang Ruan and Ming Sun^#^

State Key Laboratory of Agricultural Microbiology, College of Life Science and Technology, Huazhong Agricultural University, Wuhan, P.R. China

**^†^**Both authors contributed equally to this work.

^#^Address correspondence to Ming Sun, [m98sun@mail.hzau.edu.cn](mailto:m98sun@mail.hzau.edu.cn).

Figure S1. Sequence analysis of the 24bp *oriT* core regions before (A) and after recombination (B).


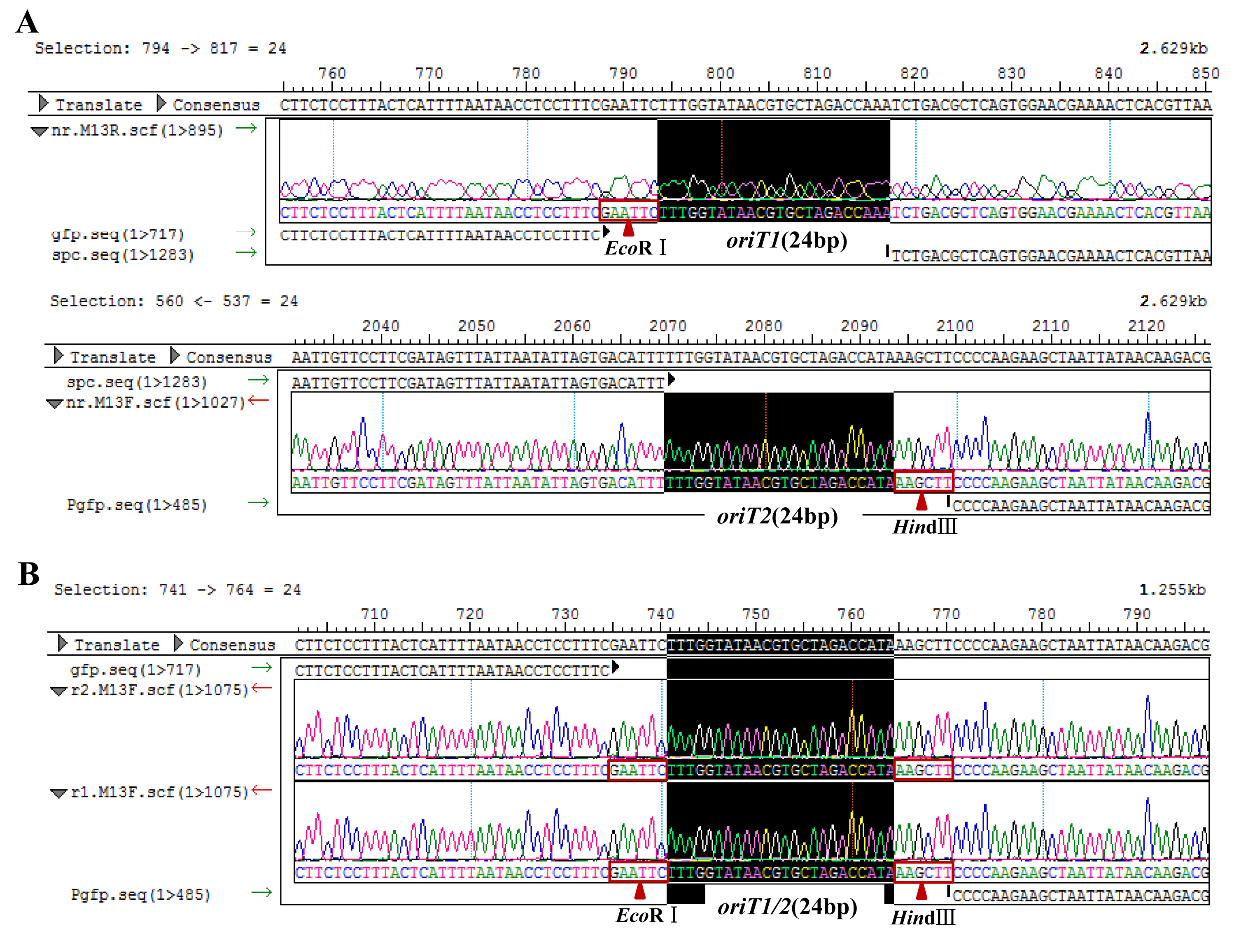


Figure S2. A test of the α-amylase activity of *amyE* mutant strain BMB0260. (A) and (B), Strain BMB171 and BMB0260 were grown on LB plates containing 1% starch; One plate (B) was stained with iodine to detect the α-amylase activity, which was indicated by the Hydrolysis circle. 1, BMB171; 2-3, BMB0260.


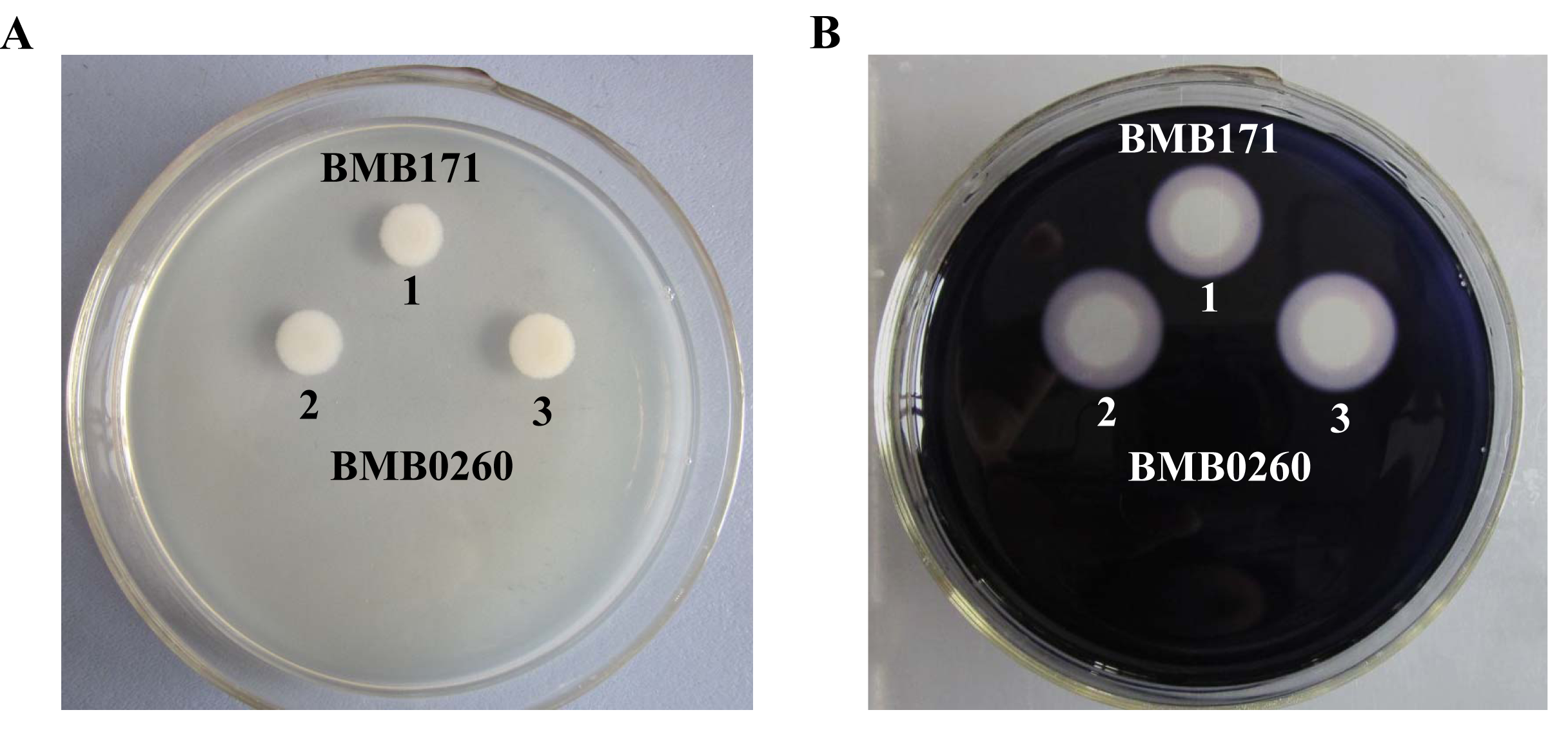


Figure S3. Cry5Ba and Cry2Aa expression in the unmarked *cry* gene integration mutants of BMB171. (A). Phase contrast microscopy graph of parasporal crystals in the unmarked *cry* gene integrate mutants. The arrows respectively indicate the diamond-shaped crystals encoded by *cry5Ba* and the round-shaped crystals encoded by *cry2Aa*. (B). SDS-PAGE analysis of crystal proteins in the unmarked *cry* gene integration mutants of BMB171. The arrows indicate the bands produced by *cry5Ba* and *cry2Aa*, respectively. 1, BMB171/*cry5Ba*-pHT304; 2-3, BMB0261; 4, YBT-1518; 5, BMB171/*cry2Aa*-pHT304; 6-7, BMB0262; 8, CT-43; 9, BMB171. M, Protein ladder.


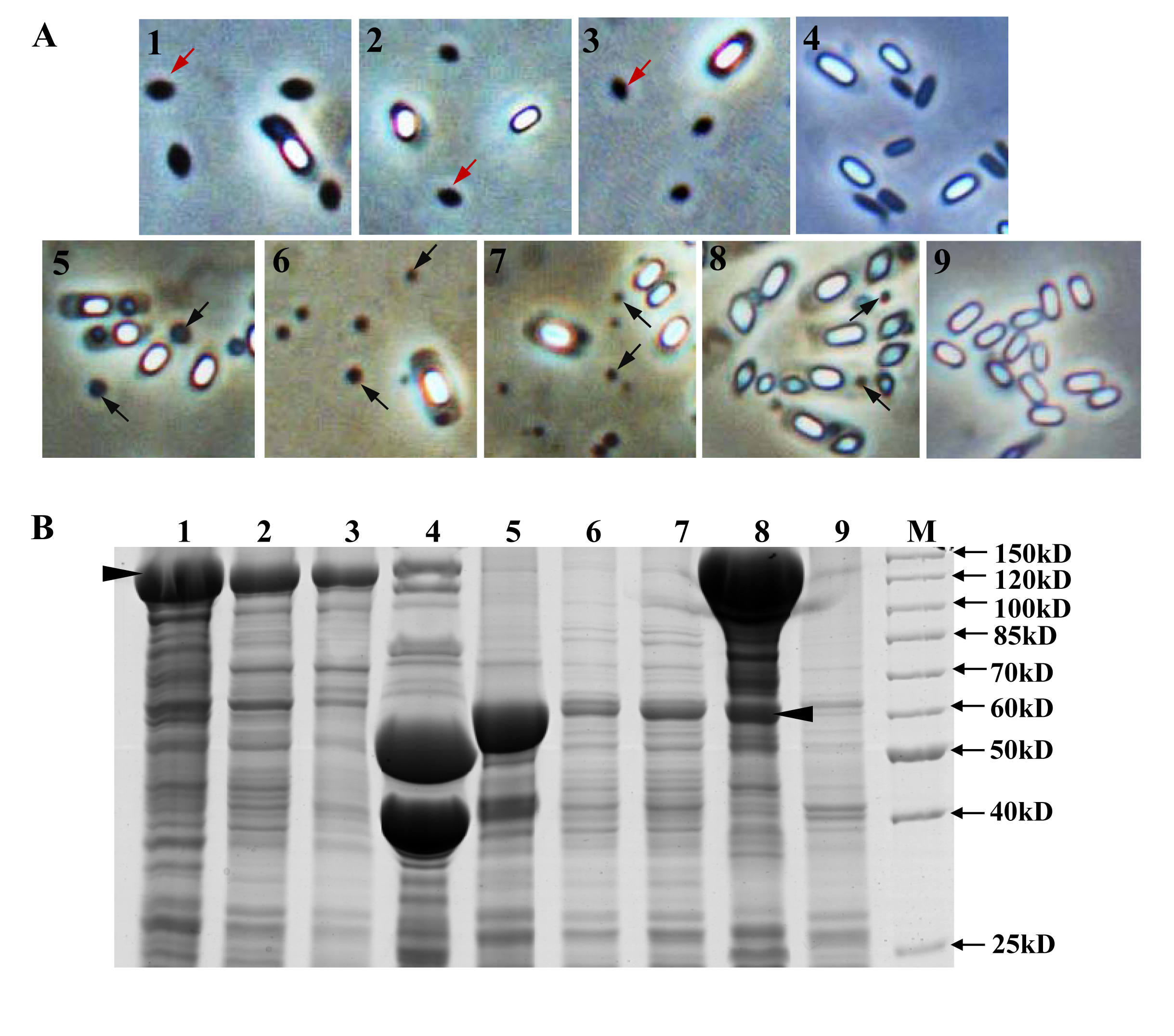

Supplement: Supplementary file 1 — 10.1186/s12934-016-0492-9 Sequence analysis of the 24bp oriT core regions before (A) and after recombination (B). Figure S2. A test of the α-amylase activity of amyE mutant strain BMB0260. (A) and (B), Strain BMB171 and BMB0260 were grown on LB plates containing 1% starch; One plate (B) was stained with iodine to detect the α-amylase activity, which was indicated by the Hydrolysis circle. 1, BMB171; 2-3, BMB0260. Figure S3. Cry5Ba and Cry2Aa expression in the unmarked cry gene integration mutants of BMB171. (A). Phase contrast microscopy graph of parasporal crystals in the unmarked cry gene integrate mutants. The arrows respectively indicates the diamond-shaped crystals encoded by cry5Ba and the round-shaped crystals encoded by cry2Aa. (B). SDS-PAGE analysis of crystal proteins in the unmarked cry gene integration mutants of BMB171. The arrows indicate the bands produced by cry5Ba and cry2Aa, respectively. 1, BMB171/cry5Ba-pHT304; 2-3, BMB0261; 4, YBT-1518; 5, BMB171/cry2Aa-pHT304; 6-7, BMB0262; 8, CT-43; 9, BMB171. M, Protein ladder. [file 12934_2016_492_MOESM1_ESM.doc]
